# Œdème aigu hémorragique du nourrisson

**DOI:** 10.11604/pamj.2019.33.267.16184

**Published:** 2019-07-29

**Authors:** Fatima-Zahra Agharbi

**Affiliations:** 1 Centre Hospitalier Régional Tétouan, Tétouan, Maroc

**Keywords:** Œdème, aigu, hémorragique, nourrisson, pseudo cocardes, edema, acute, hemorrhagic, infant, pseudo-roundel like

## Abstract

In infants less than 2 years of age, acute hemorrhagic edema of infancy (AHEI) is characterized by the rapid onset of purpuric annular lesions associated with initially local edemas of the extremities. In most cases, this disorder is benign, without visceral involvement and diagnosis is based on clinical examination. There are no specific laboratory data and the histological analysis of these lesions (which is unnecessary in the majority of cases) is usually unspecific (the patient sometimes has an unspecified leucocytoclasic vasculitis). This condition is of uncertain nosologic status, mimiking rheumatoid purpura. Sometimes the patient had had rhino-pharyngeal episode in previous days, suggesting a viral cause. There is a clear discrepancy between the good general condition and the profuse and spectacular appearance of the lesions. Children’s monitoring in the first days must be rigorous but complications are exceptional (acute intussusception'). Patient’s outcome is marked by spontaneous regression within 12 days. Treatment is based on careful monitoring of the child’s general status. Fever, purpuric lesions extension and, especially, signs of alteration of the general state may suggest the diagnosis of purpura fulminans. Another differential diagnosis is acute hemorrhagic or ecchymotic urticaria. We report the case of a 3-month old infant with diffuse pseudo-roundel like lesions in a context of apyrexy and preservation of the general state. Patient’s outcome was favorable, without receiving any treatment. This confirm the diagnosis of AHEI.

## Image en médecine

L'œdème aigu hémorragique du nourrisson (OAHN) est caractérisé par la survenue rapide de lésions purpuriques en cocardes, associées à des œdèmes initialement localisés aux extrémités chez l'enfant de moins de 2 ans. Cette affection reste le plus souvent bénigne, sans atteinte viscérale et le diagnostic est clinique, il n'existe pas de signes biologiques spécifiques et l'analyse histologique des lésions (qui est inutile dans la majorité des cas) est le plus souvent sans spécificité (il existe parfois une vascularite leucocytoclasique non spécifique). Sa place nosologique reste incertaine, faisant discuter, pour certains, une forme clinique du purpura rhumatoïde. On retrouve parfois un épisode rhino-pharyngé dans les jours précédents faisant discuter une étiologie virale. Il existe une discordance nette entre le bon état général et l'aspect profus et spectaculaires des lésions. La surveillance de ces enfants dans les premiers jours doit être rigoureuse mais les complications sont exceptionnelles (invagination intestinale aiguë?). L'évolution se fait spontanément vers la régression en une dizaine de jours. La prise en charge thérapeutique consiste en une surveillance attentive de l'état général de l'enfant. Une fièvre, l'extension des lésions purpuriques et surtout des signes d'altération de l'état général peuvent faire discuter le diagnostic de purpura fulminans. Un autre diagnostic différentiel est l'urticaire aiguë hémorragique ou ecchymotique. Nous rapportons l'observation d'un nourrisson de 3 mois qui présente des lésions pseudo-cocardes diffuses dans un contexte d'apyrexie et de conservation de l'état général. L'évolution était favorable sans aucun traitement ce qui a permis de confirmer le diagnostic d'OAHN.

**Figure 1:**
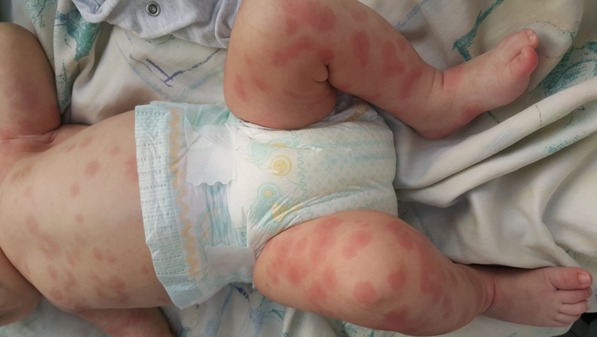
Lésions pseudo-cocardes diffuses

